# Diagnostic Accuracy of Amino Acid PET for Differentiating Tumor Recurrence or Progression from Treatment-Related Changes in Glioma Patients: A Systematic Review and Meta-Analysis

**DOI:** 10.3390/cancers18193069

**Published:** 2026-09-22

**Authors:** Małgorzata Podstawka, Anna Dębska, Natalia Koc, Samuel Chybowski, Bartosz Szmyd, Karol Zaczkowski, Ernest J. Bobeff, Paweł Ratajczyk, Bartłomiej Tomasik, Dariusz J. Jaskólski, Karol Wiśniewski

**Affiliations:** 1 Department of Neurosurgery and Neurooncology, Barlicki University Hospital, Medical University of Lodz, 90-153 Lodz, Poland; 2 Department of Anesthesiology and Intensive Therapy, Medical University of Lodz, 90-153 Lodz, Poland; 3 Department of Oncology and Radiotherapy, Faculty of Medicine, Medical University of Gdansk, 80-210 Gdansk, Poland; bartlomiej.tomasik@gumed.edu.pl; 4 Centre for Experimental Cardiooncology, Medical University of Gdansk, 80-210 Gdansk, Poland

**Keywords:** glioma, amino acid PET, recurrence, radiation necrosis, diagnostic accuracy, meta-analysis

## Abstract

**Background**: Differentiating tumor recurrence from treatment-related changes in patients with glioma remains a major diagnostic challenge because conventional MRI has limited specificity. Amino acid positron emission tomography (PET) has emerged as a promising adjunctive imaging modality. **Purpose**: To evaluate the diagnostic performance of amino acid PET for distinguishing tumor recurrence from treatment-related changes in previously treated glioma patients. **Methods**: A systematic review and meta-analysis were conducted according to the PRISMA-DTA guidelines. PubMed, Scopus, and Web of Science were searched from inception to March 2026. Studies evaluating clinically used amino acid PET tracers (MET, FET, or FDOPA) in previously treated glioma patients and reporting sufficient data to construct 2 × 2 contingency tables were included. Pooled sensitivity and specificity were estimated using a bivariate random-effects model. The primary analysis was restricted to studies reporting patient-based data from non-overlapping cohorts. A post hoc sensitivity analysis, added in response to peer review, retained both potentially overlapping patient-based datasets. Lesion- and scan-based studies were excluded from quantitative synthesis because within-patient clustering could not be accounted for using the published aggregate data. Subgroup and meta-regression analyses explored sources of heterogeneity. **Results**: Sixteen studies met the inclusion criteria. The primary synthesis comprised 12 studies reporting patient-based data from non-overlapping cohorts (728 patients, one 2 × 2 table each). Amino acid PET demonstrated a pooled sensitivity of 0.87 (95% CI 0.83–0.90) and specificity of 0.78 (95% CI 0.72–0.83). The positive likelihood ratio was 3.91 (95% CI 3.04–5.02), the negative likelihood ratio was 0.17 (95% CI 0.13–0.23), and the diagnostic odds ratio was 22.8 (95% CI 14.6–35.7). At the pooled prevalence of recurrence of 68%, a positive scan corresponded to a post-test probability of 89% and a negative scan to 27%. Univariable I^2^ estimates were 10% for sensitivity and 0% for specificity, but these measures should not be interpreted as evidence of overall homogeneity. Estimates were essentially unchanged when both potentially overlapping datasets were retained (13 studies, 756 patients). Tracer type was nominally associated with the diagnostic odds ratio (*p* = 0.044), but tracer type was strongly confounded by study sample size and publication era, and the association was not robust in the sensitivity analysis. No statistically significant subgroup differences were identified; however, these analyses were limited by the small number of studies and methodological heterogeneity. **Conclusions**: Amino acid PET usefully but not decisively revises the probability of recurrence in patients under suspicion after treatment. At the pooled pre-test probability of 68%, a positive PET increased the post-test probability of recurrence to 89%, whereas a negative PET reduced it to 27%; therefore, a negative result does not exclude recurrence. Because positivity thresholds were largely derived post hoc and reference standards were composite, these estimates are likely to be optimistic. Apparent tracer-related differences observed in subgroup and meta-regression analyses were strongly confounded by publication era and study sample size and were not supported by head-to-head within-patient comparisons. Therefore, no superiority of MET, FET, or FDOPA can be inferred from the available evidence. The analysis was limited to MET, FET, and FDOPA, and the findings may therefore not be generalizable to other amino acid tracers.

## Simple Summary

Differentiating tumor recurrence from treatment-related changes in patients with glioma remains a major diagnostic challenge. We conducted a systematic review and meta-analysis to evaluate the diagnostic accuracy of amino acid PET using MET, FET, or FDOPA for this purpose. In the primary analysis of 12 studies including 728 patients, amino acid PET showed a sensitivity of 0.87 and a specificity of 0.78, with results remaining stable in additional sensitivity analyses. Although apparent differences between tracers were observed in exploratory analyses, these were strongly confounded by publication era and study sample size. Therefore, the available evidence does not support the superiority of MET, FET, or FDOPA. These findings suggest that amino acid PET is a useful imaging tool for distinguishing tumor recurrence or progression from treatment-related changes in previously treated glioma patients, although the results should be interpreted in light of the limited number and methodological differences of the available studies.

## 1. Introduction

Gliomas are the most common primary malignant tumors of the central nervous system and are characterized by a high rate of recurrence despite multimodal treatment, including surgery, radiotherapy, and chemotherapy. Accurate differentiation between tumor recurrence and treatment-related changes, such as radiation necrosis, remains a major clinical challenge, as both conditions may present with similar radiological findings and clinical symptoms [1,2,3]. Furthermore, treatment-related changes encompass distinct pathological entities, including radiation necrosis and pseudoprogression, and variations in the definitions of these target conditions across primary studies may represent an important source of heterogeneity in the reported diagnostic performance of different imaging modalities.

Conventional magnetic resonance imaging (MRI), although widely used in clinical practice, has limited specificity for distinguishing recurrent tumor from post-treatment effects because both entities often exhibit similar imaging features, such as contrast enhancement, mass effect, and peritumoral edema [4,5,6].

Amino acid positron emission tomography (PET) has emerged as a promising functional imaging modality for evaluating gliomas. Compared with 18F-FDG, amino acid tracers demonstrate lower physiological uptake in normal brain tissue and improved tumor-to-background contrast, allowing better lesion delineation and characterization. The most commonly used tracers include 11C-methionine (MET), 18F-fluoroethyl-L-tyrosine (FET), and 18F-fluorodopa (FDOPA). Emerging approaches have also been explored, including alternative amino acid tracers such as 18F-Fluciclovine (FACBC), 18F-FAMT, and 11C-Tyrosine; however, given their comparatively limited validation, they are not considered within the scope of the present study [7,8,9,10,11,12].

Early studies demonstrated the clinical utility of MET PET for differentiating recurrent glioma from radiation necrosis, showing improved diagnostic accuracy compared with conventional imaging techniques [1,2,13,14]. Subsequent investigations confirmed these findings and highlighted the superiority of amino acid PET over FDG PET in this context [3,15,16,17].

More recent studies have expanded the role of alternative tracers, particularly FDOPA and FET, which offer practical and logistical advantages due to a longer half-life and wider availability. FDOPA PET has demonstrated high diagnostic accuracy for detecting glioma recurrence and may outperform conventional MRI in selected cases [18,19,20,21,22]. Similarly, FET PET has shown robust performance in differentiating tumor progression from treatment-related changes, particularly when advanced quantitative parameters are applied [23,24,25,26].

Recent advances in hybrid imaging and multiparametric approaches combining PET with MRI have further improved diagnostic performance. Studies incorporating dynamic PET imaging, radiomics, and multimodal analysis have demonstrated an enhanced ability to distinguish recurrence from treatment effects [27,28,29,30,31,32,33]. In addition, multicenter and larger cohort studies have confirmed the clinical utility of amino acid PET across different clinical settings [34,35,36,37,38].

Despite these advances, variability in study design, patient populations, tracer type, acquisition protocols, and quantitative thresholds has resulted in heterogeneous findings across studies. Differences in PET acquisition (static vs. dynamic), analytical methods, and reference standards may significantly influence reported diagnostic performance [22,28,30,32,34]. Therefore, a comprehensive synthesis of the available evidence is required to provide robust estimates of diagnostic accuracy and to explore potential sources of heterogeneity.

The aim of this study was to perform a systematic review and meta-analysis to evaluate the diagnostic performance of selected amino acid PET (namely MET, FET and FDOPA) for differentiating tumor recurrence from radiation necrosis or treatment-related changes in patients with previously treated glioma.

## 2. Methods

### 2.1. Study Design and Reporting Standards

This systematic review was prospectively registered in the International Prospective Register of Systematic Reviews (PROSPERO; ID: CRD420261367555). This systematic review and meta-analysis were conducted in accordance with the Preferred Reporting Items for Systematic Reviews and Meta-Analyses (PRISMA 2020) statement and the PRISMA extension for Diagnostic Test Accuracy (PRISMA-DTA). The methodological approach followed established recommendations for meta-analyses of diagnostic accuracy studies. The study protocol was developed a priori to minimize the risk of bias.

### 2.2. Inclusion Criteria

Studies were included if they met all of the following criteria:

1. Original research articles evaluating patients with previously treated glioma.
2. Use of amino acid PET to differentiate tumor recurrence from radiation necrosis or treatment-related changes.
3. Availability of sufficient data to construct a 2 × 2 contingency table.
4. Histopathological confirmation of glioma diagnosis, classified according to the criteria applicable at the time of the original study, and/or predefined clinical–radiological follow-up.
5. Full-text articles published in English.

### 2.3. Exclusion Criteria

Studies were excluded if they met any of the following criteria:

1. Reviews, meta-analyses, editorials, letters, or conference abstracts without sufficient data.
2. Case reports or small case series with fewer than 10 patients.
3. Animal or preclinical studies.
4. Studies without extractable diagnostic accuracy data.
5. Studies with overlapping patient populations (in such cases, the most comprehensive or most recent dataset was included). Studies from the same research group were carefully assessed for potential overlap and were included when they provided distinct or independently analyzable diagnostic datasets.

### 2.4. Literature Search

A comprehensive literature search was performed in PubMed, Scopus and Web of Science from database inception to March 2026. No initial restrictions on publication year were applied. The search was limited to articles in English. To minimize the risk of missing recently published evidence, records published between January and March 2026 were additionally reviewed for potential eligibility. Reference lists of the included studies and relevant reviews were screened for additional eligible studies using backward citation tracking. Forward citation tracking was not performed. Clinical trial registries and gray literature sources were not systematically searched.

The search strategy combined terms related to glioma, positron emission tomography, amino acid tracers, and recurrence or radiation necrosis.

The database search strategy was as follows:

((glioma) OR (brain tumor) OR (brain neoplasm)) AND ((positron emission tomography) OR PET) AND ((FET) OR (MET) OR (FDOPA) OR (amino acid PET) OR (methionine) OR (fluoroethyltyrosine) OR (fluorodopa)) AND ((recurrence) OR (tumor progression) OR (radiation necrosis) OR (treatment-related changes)).

In addition, reference lists of included articles and relevant reviews were manually screened to identify further eligible studies. When necessary, corresponding authors were contacted to obtain missing or unclear data.

### 2.5. Study Selection

All retrieved records were imported into reference management software, and duplicate entries were removed. Two reviewers independently screened titles and abstracts to exclude clearly irrelevant studies. Full-text articles were subsequently assessed for eligibility according to predefined criteria. Any disagreements were resolved through discussion and consensus or, when necessary, consultation with a third reviewer. The study selection process was documented using a PRISMA flow diagram (Figure 1). Prisma checklist and study-level diagnostic datasets of the research included in the quantitative analysis can be found in the Supplementary Materials (Prisma Checklist, Supplementary Table S1). Two reports from the same research group [17,18] were examined in detail for cohort overlap. Both were prospective single-center series from the same department, published within five weeks of each other, evaluating the same tracer against the same composite reference standard, with 28 and 35 patients, respectively and closely similar age and sex distributions; neither report states the recruitment period, and the authors could not be reached for clarification. The smaller series could not be shown to be independent of the larger and was therefore treated as an overlapping cohort. Because individual-level patient data and recruitment dates were unavailable, overlap between the two cohorts could not be confirmed directly and was inferred from the published cohort characteristics. In accordance with the predefined rule, only the larger and more recent dataset [18] was retained in the primary quantitative synthesis. Sensitivity Analysis A retained both datasets to evaluate the robustness of the pooled estimates to potential misclassification of their overlap status.

### 2.6. Data Extraction

Data extraction was performed independently by two reviewers using a predefined standardized form. Extracted variables included study characteristics (first author, year of publication, country, and study design), patient characteristics (number of patients and/or lesions, age, tumor type and grade), imaging details (type of amino acid tracer, PET acquisition protocol, static or dynamic imaging, quantitative parameters and cutoff values), reference standard, and diagnostic performance data (true positives, false positives, true negatives, and false negatives). For the reference standard, we additionally extracted the verification method (histopathology, clinical–radiological follow-up, or a composite of both) and the reported duration or minimum criterion for follow-up, where available.

Exactly one 2 × 2 table was extracted per study. Where several cutoff values were reported, the threshold designated optimal by the original authors was used. In most studies, the threshold was optimized and evaluated within the same dataset, making the reported accuracy susceptible to overfitting and optimism bias relative to the performance of a prespecified threshold in an independent cohort. No analytical correction for this exists, and the direction and approximate magnitude of the resulting bias are addressed in the Discussion. Where the number of entries in the 2 × 2 table differed from the number enrolled, the former was used. Patient-based data were extracted in preference to lesion-based or scan-based data whenever both were reported.

The unit of analysis was classified as patient-based, lesion-based or scan-based. This restriction, and the exclusion of the overlapping cohort described above, were not part of the registered protocol (PROSPERO 1367555); both were introduced after peer review and are identified as post hoc throughout.

### 2.7. Quality Assessment

The methodological quality of included studies was assessed using the Quality Assessment of Diagnostic Accuracy Studies-2 (QUADAS-2) tool. Four domains were evaluated: patient selection, index test, reference standard, and flow and timing. Each domain was assessed for risk of bias, and the first three domains were additionally evaluated for concerns regarding applicability. Quality assessment was performed independently by two reviewers. Discrepancies were resolved by consensus. The results were summarized in both tabular and graphical formats.

### 2.8. Statistical Analysis

The primary outcomes were the summary sensitivity and specificity of amino acid PET for differentiating tumor recurrence or progression from treatment-related changes. The primary analysis was restricted to the 12 studies reporting patient-based data from non-overlapping cohorts (728 patients). A post hoc sensitivity analysis retained all 13 patient-based datasets, including both potentially overlapping Karunanithi datasets (756 patients), to assess the robustness of the pooled estimates to uncertainty regarding their overlap status. Lesion- and scan-based datasets were excluded from quantitative synthesis because within-patient clustering could not be accounted for using the available aggregate data. Sensitivity and specificity were pooled jointly using a bivariate random-effects model fitted by restricted maximum likelihood on the logit scale. A continuity correction of 0.5 was added to all four cells of studies containing a zero cell, and to those studies only. The between-study correlation between logit sensitivity and logit specificity was not estimable in these data: unconstrained estimation converged to the parameter boundary and the profile restricted log-likelihood was essentially flat, changing by less than 0.05 across the range from −0.95 to +0.95. The correlation was therefore constrained to zero in the model reported here, and the consequences are addressed in the limitations. Positive and negative likelihood ratios and the diagnostic odds ratio were derived analytically from the same fitted summary point, so that all reported measures are mutually consistent by construction; their confidence intervals were obtained by the delta method from the covariance matrix of the summary estimates. Post-test probabilities were calculated from the summary positive and negative likelihood ratios using Bayes’ theorem across pre-test probabilities from 10% to 90% in 10-percentage-point increments. This range was chosen to illustrate test performance across lower- and higher-risk clinical settings and encompasses the prevalence observed in the included studies. Because the between-study correlation was not estimable, a regression-based summary receiver operating characteristic (SROC) curve was not fitted. The summary point is instead presented with its 95% confidence region and 95% prediction region, together with a symmetric SROC curve of constant diagnostic odds ratio. The latter is an approximation, is labeled as such in the figure, and the area under it is reported only for comparability with earlier reviews; it is not an estimate derived from the fitted bivariate model. For individual studies, 95% confidence intervals for sensitivity and specificity were calculated by the Wilson score method, used consistently in all tables and all figures. Univariable random-effects models on the logit scale (DerSimonian–Laird) provided I^2^, τ^2^ and Cochran’s Q separately for sensitivity and specificity. These statistics describe variation in each measure separately and do not capture a threshold effect; they are reported with that caveat. Publication bias was assessed by regressing the log diagnostic odds ratio on the inverse square root of the effective sample size, weighted by the effective sample size (Deeks’ test), with *p* < 0.10 taken to indicate asymmetry; this test has low power with fewer than 20 studies. Robustness was examined by leave-one-out refitting of the bivariate model. Because zero cells were present in four studies in the primary dataset, robustness to the continuity correction was additionally assessed in a post hoc sensitivity analysis using a one-stage binomial bivariate random-effects model fitted directly to the study-level true-positive, false-negative, true-negative, and false-positive counts. This model uses the binomial likelihood and therefore requires no continuity correction. To isolate the effect of zero-cell handling from the separate issue of correlation identifiability, the between-study correlation was constrained to zero as in the primary model. A further post hoc sensitivity analysis excluded studies rated as a high risk of bias in at least one QUADAS-2 domain. The remaining studies were refitted using the same bivariate random-effects specification as in the primary analysis. Tracer type was examined by subgroup analysis and by univariable mixed-effects meta-regression on the log diagnostic odds ratio using the Knapp–Hartung adjustment, which is appropriate when few studies are available. Bubble plots were used to visualize these associations, with bubble size proportional to study weight. Study design could not be examined, because after exclusion of the overlapping Karunanithi dataset only one prospective study remained. Subgroup analyses by reference standard, acquisition protocol (static versus dynamic) and tumor grade were not performed, because these characteristics were reported too inconsistently across studies to permit meaningful classification.

All analyses were performed in Python 3.14.3 using numpy 2.4.3, scipy 1.17.1 and statsmodels 0.14.6; figures were produced with matplotlib 3.10.9. The bivariate model was fitted by direct restricted maximum likelihood optimization of the two-stage approximate likelihood. The complete analysis script and the study-level 2 × 2 data are provided as Supplementary Material, so that every estimate, figure and test reported in this article can be reproduced exactly from the raw counts (Complete Code Analysis).

## 3. Results

The literature search identified a total of 1718 records, including 827 from PubMed, 838 from Scopus, and 50 from Web of Science and three identified through snowballing. After the removal of 573 duplicates, 1145 records remained for title and abstract screening. During title and abstract screening, 1012 records were excluded. The study selection process is summarized in Figure 1 (PRISMA flow diagram). Of the 133 full-text reports assessed, 117 were excluded: 54 did not provide sufficient data to construct a 2 × 2 table for amino acid PET alone, most commonly because results were reported only for combined PET/MRI, radiomic or multiparametric models; 21 had an ineligible population; 14 were not original studies; 11 used an inappropriate or unclear reference standard; 9 reported overlapping cohorts; 5 included fewer than 10 patients; and 3 were excluded for other reasons. Sixteen studies were included in the systematic review, and 12 of these formed the primary quantitative synthesis (Figure 1).

### 3.1. Risk of Bias Assessment

Risk of bias was assessed using the QUADAS-2 tool across four domains: patient selection, index test, reference standard, and flow and timing (Table 1). Overall, the methodological quality of the included studies was moderate. The main concerns were identified in the domains of patient selection and index test. In the patient selection domain, the majority of studies (14) were retrospective and included selected cohorts of patients with suspected recurrence after treatment, which may have introduced selection bias. Only two of the studies were clearly prospective or reported consecutive patient inclusion, resulting in predominantly unclear or high risk of bias in this domain. For the index test, the risk of bias was frequently rated as unclear due to insufficient reporting of whether PET interpretation was performed blinded to the reference standard. In addition, threshold values for PET positivity (e.g., TBR or SUV cutoffs) were often determined post hoc or not prespecified, increasing the likelihood of data-driven threshold selection. In the reference standard domain, most studies used histopathology and/or clinical–radiological follow-up as the reference standard. While this approach is commonly accepted in neuro-oncology, the combination of different reference standards and variability in follow-up duration contributed to an unclear risk of bias in several studies. Regarding flow and timing, the risk of bias was generally low to moderate. However, in some studies, incomplete reporting of the interval between PET imaging and reference standard assessment or exclusion of patients from the final analysis led to an unclear risk. Concerns regarding applicability were low across all domains, as all included studies evaluated previously treated glioma patients using amino acid PET to differentiate tumor recurrence from treatment-related changes.

### 3.2. Quantitative Results

Sixteen studies met the inclusion criteria. Three reported diagnostic accuracy on a lesion or scan basis rather than per patient (Sonoda, 12 scans; Shishido, 21 lesions; Henriksen, 76 lesions), so that a single patient could contribute several correlated observations. A further dataset, Karunanithi 2013a [17] (28 patients), was considered potentially overlapping with Karunanithi 2013b [18] (35 patients). Both were single-center prospective series from the same department, published within five weeks of one another, using the same tracer, index test and composite reference standard, with closely similar age and sex distributions. Because individual-level data and recruitment periods were unavailable, the extent of overlap could not be verified; however, the smaller series was compatible with being partly or wholly included in the larger cohort. In accordance with the protocol rule that the most comprehensive dataset is retained when cohorts overlap, only Karunanithi 2013b [18] was carried forward. The primary quantitative synthesis therefore comprises 12 studies and 728 independent patients, each contributing exactly one 2 × 2 table, as detailed in the Supplementary Materials. The three lesion- or scan-based studies were retained in the qualitative systematic review but excluded from all quantitative analyses because the available aggregate data did not permit adjustment for within-patient clustering.

All included studies enrolled patients with clinical or radiological suspicion of recurrence, but the target condition was not defined uniformly. Six studies explicitly contrasted tumor recurrence with radiation necrosis [1,2,3,7,13,35], two contrasted recurrence with treatment-related changes in general [23,33], and the remaining eight reported diagnostic accuracy without specifying which non-recurrence condition was being distinguished [14,15,17,18,19,25,29,36]. Radiation necrosis, non-specific treatment-related changes, and pseudoprogression are not the same entity and are unlikely to have identical imaging behavior; pooling them means the summary estimates describe a composite target condition rather than a single one. This heterogeneity of the target condition could not be examined quantitatively, because most studies did not report outcomes separately by underlying pathology.

In the primary analysis of 12 patient-based, non-overlapping studies (728 patients), the summary sensitivity was 0.87 (95% CI 0.83–0.90) and the summary specificity was 0.78 (95% CI 0.72–0.83). The positive likelihood ratio was 3.91 (95% CI 3.04–5.02), the negative likelihood ratio was 0.17 (95% CI 0.13–0.23) and the diagnostic odds ratio was 22.8 (95% CI 14.6–35.7). All four measures were derived from the same fitted summary point and are therefore mutually consistent (Table 2).

Univariable heterogeneity estimates were I^2^ = 10.3% for sensitivity (τ^2^ = 0.028; Cochran’s Q = 12.26, df = 11, *p* = 0.34) and I^2^ = 0% for specificity (τ^2^ = 0.000; Q = 6.63, df = 11, *p* = 0.83). These statistics describe marginal between-study variation in sensitivity and specificity separately and should not be interpreted as evidence of overall homogeneity. They do not capture threshold effects or the substantial clinical and methodological differences between studies. In the bivariate model, the between-study standard deviation was 0.23 on the logit sensitivity scale and zero on the logit specificity scale, while the between-study correlation was not estimable; therefore, a threshold effect can be neither demonstrated nor excluded. The wide scatter of individual specificity, which ranges from 0.60 to 1.00, is attributable to sampling error in small denominators rather than to genuine between-study variation: eight of the twelve studies contributed fewer than 20 patients without recurrence (range 5–16) (Figure 2 and Figure 3).

In receiver operating characteristic space the summary point lay at a false-positive rate of 0.22 and a sensitivity of 0.87. Because the between-study correlation was not estimable, no regression-based SROC curve was fitted; the area under a symmetric curve of constant diagnostic odds ratio was 0.90, which is reported only as an approximation for comparability with earlier reviews. The 95% prediction region was appreciably wider than the confidence region in the sensitivity dimension and no wider in the specificity dimension, reflecting the absence of detectable between-study variation in specificity (Figure 4).

Every included study defined test positivity using a different quantitative metric or a different cut-off, and in most studies that cut-off was identified post hoc within the same dataset in which it was evaluated. The summary estimates therefore describe the performance of amino acid PET at study-specific, data-derived thresholds, and are expected to be optimistic relative to what would be obtained with a single prespecified threshold applied to a new cohort. This may inflate pooled diagnostic accuracy and reduce the external validity of the summary estimates. This is a limitation of the evidence base rather than of the synthesis, and it cannot be removed analytically.

Deeks’ test did not detect funnel-plot asymmetry (slope +6.36, *p* = 0.21). With only 12 studies this test has very low power, so it does not provide meaningful reassurance against small-study effects, and the possibility of publication bias cannot be excluded (Figure 5).

### 3.3. Sensitivity Analysis

Leave-one-out refitting of the bivariate model did not identify any influential study. Across the twelve refits, the summary sensitivity ranged from 0.85 to 0.88 and the summary specificity from 0.77 to 0.79; the largest single shift was produced by the omission of Puranik 2025 [36], the largest cohort, which lowered sensitivity from 0.87 to 0.85.

A post hoc sensitivity analysis assessed uncertainty regarding the potential overlap of the two Karunanithi datasets. Retaining both datasets (13 patient-based studies, 756 patients) gave a summary sensitivity of 0.87 (95% CI 0.83–0.90) and specificity of 0.78 (95% CI 0.72–0.83), with a diagnostic odds ratio of 23.6 (95% CI 15.1–36.8). These estimates were virtually unchanged from the primary analysis, indicating that uncertainty regarding the overlap status of the two cohorts did not materially affect the principal findings. Four of the twelve studies in the primary analysis contained at least one zero cell. In a one-stage binomial bivariate sensitivity analysis fitted directly to the study-level counts without continuity correction, summary sensitivity was 0.883 (95% profile-likelihood CI 0.845–0.924) and summary specificity was 0.794 (95% profile-likelihood CI 0.739–0.850). The corresponding positive and negative likelihood ratios were 4.29 and 0.15, respectively, and the diagnostic odds ratio was 29.1. Compared with the primary two-stage model, sensitivity and specificity increased by only 0.016 in absolute terms, indicating that the use of the 0.5 continuity correction did not materially affect the principal findings. When the between-study correlation was estimated freely in the one-stage binomial model, the estimate again approached the parameter boundary (ρ = 0.999), indicating weak identifiability of the covariance structure. Across fixed correlations from −0.95 to +0.95, summary sensitivity varied only from 0.883 to 0.885 and specificity from 0.794 to 0.796. Thus, although the correlation parameter itself could not be estimated reliably, the pooled sensitivity and specificity were essentially insensitive to the imposed zero-correlation assumption. A further sensitivity analysis excluded the six primary-analysis studies rated as high risk of bias in at least one QUADAS-2 domain. The remaining six studies comprised 374 patients. Summary sensitivity was 0.893 (95% CI 0.821–0.938) and specificity was 0.810 (95% CI 0.723–0.874), compared with 0.867 and 0.778, respectively, in the primary analysis. The corresponding PLR was 4.70, NLR was 0.13, and DOR was 35.7 (95% CI 16.5–77.3). Thus, the exclusion of high-risk studies did not attenuate the estimated diagnostic performance, although precision was reduced because only six studies remained. The primary and sensitivity analyses are compared in Figure 6 and Table 3.

The corresponding forest plots and SROC curve for the patient-based sensitivity analysis are presented in Figure 6.

### 3.4. Subgroup Analysis

Subgroup analysis by tracer type was performed to explore potential sources of heterogeneity, including tracer type and study design. The results of subgroup analyses are summarized in Table 4.

When stratified by tracer, MET PET gave the highest estimates (sensitivity 0.89, 95% CI 0.80–0.95; specificity 0.82, 95% CI 0.67–0.91; k = 5, 130 patients), FET PET intermediate estimates (0.87, 0.79–0.92; and 0.80, 0.71–0.86; k = 4, 347 patients) and FDOPA PET the lowest (0.84, 0.78–0.89; and 0.73, 0.62–0.82; k = 3, 251 patients). These subgroups differ systematically in more than tracer, as set out below.

No subgroup analysis by study design was performed. After exclusion of the overlapping Karunanithi 2013a dataset [17], a single prospective study remained, and a subgroup of one cannot support a comparison. Subgroup analyses by reference standard, acquisition protocol (static versus dynamic) and tumor grade were also not performed: fifteen of the sixteen studies used a composite of histopathology and clinical–radiological follow-up without reporting the proportion verified by each route, and acquisition protocol and grade distribution were reported too inconsistently to permit classification. Molecular subtype, prior treatment regimen, and the tumor classification criteria used were likewise reported inconsistently across the primary studies. Moreover, the included studies span several WHO classification eras, limiting retrospective harmonization of tumor entities. Consequently, these variables could not be classified with sufficient consistency to support meaningful subgroup or sensitivity analyses. These sources of clinical and methodological heterogeneity may contribute to between-study differences, but their effects cannot be examined reliably using the available study-level data.

### 3.5. Meta-Regression Analysis

To further explore potential sources of heterogeneity beyond subgroup comparisons, meta-regression analysis was performed to investigate the impact of tracer type (FET vs. MET vs. FDOPA). A bubble plot was used to visualize the relationship between tracer type and diagnostic performance (Figure 7). Meta-regression by study design was not feasible for the reason noted above.

In univariable mixed-effects meta-regression on log(DOR) with the Knapp–Hartung adjustment, tracer type was nominally associated with diagnostic performance (omnibus F(2,9) = 4.51, *p* = 0.044). Relative to MET, the log diagnostic odds ratio was lower for FDOPA (β = −1.42, 95% CI −2.60 to −0.23, *p* = 0.025) and not clearly different for FET (β = −0.63, 95% CI −1.80 to +0.55, *p* = 0.258). These study-level associations are exploratory and cannot be interpreted as tracer effects. Tracer type was strongly confounded with publication era and study sample size, and no within-patient head-to-head comparisons were available. The five MET studies were published between 2004 and 2017 and contributed 130 patients in total (median 29 per study), whereas the FDOPA and FET studies were published from 2013 onwards and contributed 251 and 347 patients (median 106 and 66). Smaller and older diagnostic accuracy studies characteristically report more extreme performance, and with 12 studies and no head-to-head comparisons, the design cannot separate these explanations. The result is reported for transparency only. In the sensitivity analysis retaining both potentially overlapping Karunanithi datasets, the same contrast did not reach conventional significance (*p* = 0.098 with 13 studies), further indicating that the finding is not robust.

Taken together, the moderator analyses are uninformative about the questions that matter clinically. With 12 studies, no head-to-head comparisons, complete confounding between tracer and study era, and no usable information on acquisition protocol, positivity threshold, reference standard or tumor grade, this dataset cannot establish which tracer performs better, nor can it establish that the tracers are equivalent. Both conclusions would be unsupported.

## 4. Discussion

In this systematic review and meta-analysis, we demonstrated that amino acid PET provides high diagnostic accuracy for differentiating tumor recurrence from treatment-related changes in patients with previously treated glioma. Across 12 non-overlapping patient-based cohorts, the summary sensitivity of 0.87 and a specificity of 0.78 indicate that this imaging modality yields good overall diagnostic performance in a clinically challenging scenario where conventional imaging remains limited.

Restricting the primary analysis to patient-based, non-overlapping datasets was a deliberate choice. Pooling patients, lesions and repeated scans as if they were independent observations understates uncertainty, and retaining two datasets from the same center that plausibly describe the same patients double-counts them. These estimates are consistent with earlier reviews. A central finding of this review is that the available evidence does not permit a reliable comparison of the relative diagnostic performance of MET, FET and FDOPA. The question of whether the three tracers perform differently, however, cannot be answered from this evidence base, and we wish to be explicit about why. Small and early diagnostic accuracy studies systematically report more favorable performance, and with 12 studies, this design cannot distinguish a tracer effect from a study-size or publication-era effect. It would be equally wrong to conclude that MET is superior and to conclude that the tracers are equivalent: the absence of a robust difference reflects the limits of the available evidence, not established similarity. Tracer-specific transport mechanisms and kinetic profiles could plausibly produce genuine differences [17,18,19,20,21], and only within-patient head-to-head comparison can establish whether they do.

The influence of study design could not be assessed at all. Only two of the sixteen studies were prospective; both were from the same department and used the same tracer, and on re-examination, they were judged to describe overlapping cohorts. A single prospective dataset therefore remains, and this literature currently provides no basis for comparing prospective with retrospective evaluations of amino acid PET.

Low statistical heterogeneity should not be mistaken for methodological homogeneity [21,27,31,33,34]. The included studies differed in patient selection, imaging protocol, positivity threshold, reference standard, glioma grade, molecular characterization, prior treatment regimens, and the tumor classification framework applicable at the time of diagnosis. Several of these characteristics, particularly molecular features, treatment regimens, and tumor classification criteria, were incompletely and inconsistently reported and could not be harmonized reliably across studies. Consequently, their potential contribution to between-study heterogeneity could not be examined quantitatively.

The clinical value of these estimates is best judged in absolute terms. Recurrence was confirmed in 68% of the pooled population (range across studies 40–78%), reflecting cohorts selected for clinical or radiological suspicion of recurrence. At that pre-test probability, a positive amino acid PET raises the probability of recurrence from 68% to about 89%, and a negative scan lowers it to about 27%. Across a broader range of pre-test probabilities, the corresponding post-test probabilities varied substantially. At pre-test probabilities of 20%, 40%, 60%, and 80%, a positive PET increased the probability of recurrence to approximately 49%, 72%, 85%, and 94%, respectively, whereas a negative PET reduced it to approximately 4%, 10%, 20%, and 41%, respectively. Amino acid PET is therefore a useful, but not a decisive test: roughly one in four negative scans still represents recurrence, and the frequent description of amino acid PET as a means of ruling out recurrence overstates what these data support. In populations with a lower prevalence of true recurrence the positive predictive value would fall further. Thus, particularly in patients with a high pre-test probability, a negative PET cannot reliably exclude recurrence. The test should be used to modify, not to replace, clinical and MRI-based judgment.

Conventional MRI alone showed low diagnostic accuracy (pooled sensitivity and specificity of 68% (95%CI 51–81) and 77% (45–93), respectively) in distinguishing tumor recurrence from treatment-related changes. Advanced MRI techniques, such as perfusion MRI and spectroscopy MRI, showed more promise (pooled sensitivity 87% (82–91) and 91% (79–97) for dynamic susceptibility perfusion MRI and spectroscopy MRI, respectively) [4]. Nevertheless, studies comparing advanced MRI techniques, especially perfusion MRI, with amino acid PET have produced somewhat heterogeneous results. Several investigations reported comparable diagnostic performance between the two modalities for differentiating recurrent glioma from treatment-related changes [28,31]. In contrast, other studies have suggested superior diagnostic performance for amino acid PET. Jabeen et al. reported that amino acid PET achieved higher accuracy than individual MRI parameters, although the combination of PET and MRI further improved diagnostic confidence, while Harat et al. demonstrated that integrated amino acid PET and MRI analyses more accurately identified malignant tumor tissue than MRI alone [37,38]. Future research should prioritize well-designed, prospective head-to-head comparative studies to directly evaluate the diagnostic performance of amino acid PET against advanced MRI modalities, including perfusion MRI and MR spectroscopy, given the heterogeneous findings reported in the current literature.

Advances in imaging methodology have enhanced the diagnostic capabilities of amino acid PET. Dynamic acquisition protocols, radiomics, and hybrid PET/MRI approaches have shown promising results in improving differentiation between tumor progression and treatment-related changes [26,27,28,29,30,31,32]. In particular, dynamic FET and FDOPA PET parameters, as well as multiparametric models incorporating MRI-derived metrics, have demonstrated improved diagnostic performance compared with single-parameter analyses. Larger and multicenter studies have also confirmed the clinical utility of amino acid PET across diverse patient populations and clinical settings [33,34,35,36].

An additional important consideration is the role of amino acid PET in surgical planning and its limitations in translating preoperative imaging findings into intraoperative decision-making. Amino acid PET has been increasingly used to delineate metabolically active tumor regions beyond contrast-enhancing areas on MRI, potentially enabling more accurate targeting of biopsy and guiding the extent of resection. Several studies have demonstrated that PET-defined tumor margins may extend beyond conventional MRI boundaries, particularly in high-grade gliomas, thereby providing complementary information for surgical planning [14,16,28,29,38]. However, the potential role of amino acid PET in surgical planning and biopsy targeting should be considered a secondary narrative consideration, as the included studies primarily assessed diagnostic accuracy rather than surgical outcomes.

The integration of amino acid PET into intraoperative workflows remains challenging. Spatial discrepancies between preoperative PET imaging and intraoperative anatomy, due to factors such as brain shift, limit the direct applicability of PET-defined targets during surgery. Furthermore, variability in tracer uptake, threshold definitions, and the lack of standardized segmentation methods may affect the reproducibility of PET-based tumor delineation [21,27,30]. While hybrid PET/MRI and neuronavigation systems may partially mitigate these limitations, the translation from preoperative metabolic imaging to real-time surgical guidance remains imperfect.

Consequently, amino acid PET should be considered a complementary tool rather than a standalone modality for treatment decision-making. Its greatest value may lie in preoperative planning, biopsy guidance, and the identification of biologically aggressive tumor subregions, while intraoperative interpretation should be performed in conjunction with anatomical imaging and surgical expertise.

The review has three strengths. It was conducted and reported according to PRISMA-DTA, with a prospectively registered protocol, a search of three databases, and duplicate screening and extraction. The primary synthesis uses one independent patient-based 2 × 2 table per study, avoiding both within-patient clustering and duplicate cohorts. Finally, the study-level counts and the complete analysis code are provided so that every number in this article can be recomputed by a reader from the raw data.

The limitations are substantial and constrain how these estimates should be used. First, and most importantly, the summary estimates describe performance at study-specific thresholds that were in most cases selected post hoc within the same dataset in which they were evaluated. This introduces overfitting and optimism bias and may inflate the pooled sensitivity, specificity, and diagnostic odds ratio. Accordingly, the pooled estimates should be interpreted as an upper-bound estimate of performance rather than as the expected accuracy of a single prespecified threshold applied prospectively to an independent cohort. Second, fifteen of the sixteen studies used a composite reference standard incorporating histopathology and clinical–radiological follow-up, often without reporting how many patients were verified by each route. The use of different verification methods within the same study may introduce differential verification bias. Moreover, where the index PET findings contributed to clinical–radiological or multidisciplinary determination of the final diagnosis, incorporation bias may also have occurred. Variability and incomplete reporting of follow-up duration further limit the comparability of reference standards across studies. Furthermore, most studies grouped radiation necrosis, pseudoprogression, and other treatment-related changes into a single category despite their distinct biological characteristics, precluding separate assessment of diagnostic performance according to the underlying pathology. The magnitude and direction of these biases cannot be quantified reliably from the published data. Third, all cohorts were selected for suspected recurrence, giving a pooled prevalence of 68%; the predictive values reported above do not transfer to unselected surveillance populations. Fourth, eleven of the twelve studies in the primary analysis were retrospective and most were single-center, with high or unclear risk of bias for patient selection and index test in the majority. Exclusion of studies rated as high risk of bias in any QUADAS-2 domain did not reduce the summary sensitivity or specificity, although the resulting estimates were less precise because only six studies remained. Fifth, with twelve studies, the moderator analyses have little power, and Deeks’ test is close to uninformative. With only twelve studies, eight of which included fewer than 20 patients without recurrence, the between-study correlation and heterogeneity in specificity were weakly identified. A one-stage binomial analysis produced closely similar summary sensitivity and specificity, and these estimates changed negligibly when the correlation was fixed across a wide range of values. Nevertheless, the covariance structure itself could not be estimated reliably, and this uncertainty should be considered when interpreting between-study heterogeneity and the shape of the summary ROC relationship. Sixth, individual-level patient data and recruitment dates were unavailable for the two Karunanithi studies. Consequently, their classification as overlapping cohorts was inferred from similarities in the published cohort descriptions and could not be independently verified. Misclassification of their overlap status therefore remains possible. However, Sensitivity Analysis A, in which both datasets were retained, produced virtually unchanged pooled estimates, indicating that this uncertainty did not materially influence the principal findings. Finally, only three tracers in routine clinical use were considered, and the findings do not extend to other amino acid tracers, to dynamic or radiomic parameters, or to combined PET/MRI models, all of which were excluded because they could not be reduced to a single 2 × 2 table. Furthermore, the relatively small number of studies in certain subgroups, particularly prospective studies, limits the strength of subgroup comparisons. Another limitation is that clinical trial registries and gray literature sources were not systematically searched. Consequently, unpublished studies, conference abstracts, dissertations, or other non-indexed evidence may have been missed. This may have introduced publication bias, as studies reporting statistically significant or positive findings are more likely to be published and indexed in major bibliographic databases, whereas studies with negative or inconclusive results may remain unpublished.

Future research should focus on prospective, multicenter studies with standardized imaging protocols and predefined quantitative thresholds. The integration of amino acid PET with advanced MRI techniques and the application of artificial intelligence and radiomics approaches may further improve diagnostic accuracy and clinical decision-making.

## 5. Conclusions

In 12 non-overlapping patient-based cohorts comprising 728 patients, amino acid PET distinguished tumor recurrence or progression from treatment-related changes with a summary sensitivity of 0.87 and a specificity of 0.78. In cohorts selected for suspected recurrence, this shifts the probability of recurrence from 68% before the scan to 89% after a positive result and to 27% after a negative one: a clinically useful, but not decisive, change. These estimates were obtained at study-specific, largely post hoc thresholds and under composite reference standards, and are therefore likely to be optimistic. The data do not support any claim about the relative or equivalent performance of MET, FET and FDOPA, because tracer type is completely confounded with study size and publication era, and no head-to-head comparisons exist. What the field needs is not further retrospective single-center series, but prospective multicenter studies with a threshold fixed in advance, a uniform and independently applied reference standard, patient-level reporting, and a direct comparison of tracers within the same patients.

## Figures and Tables

**Figure 1:**
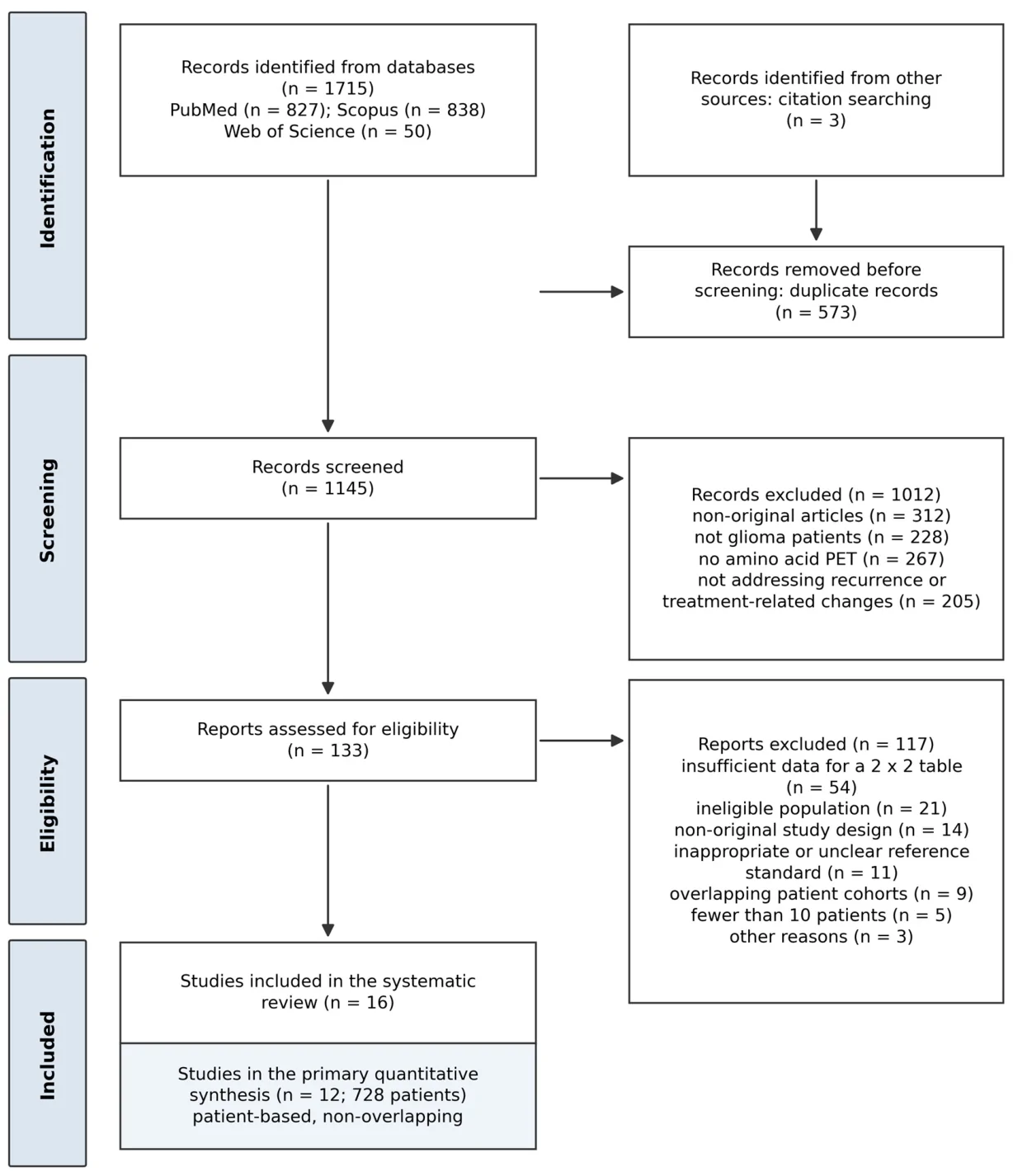
The flowchart of publication selection process [39].

**Figure 2:**
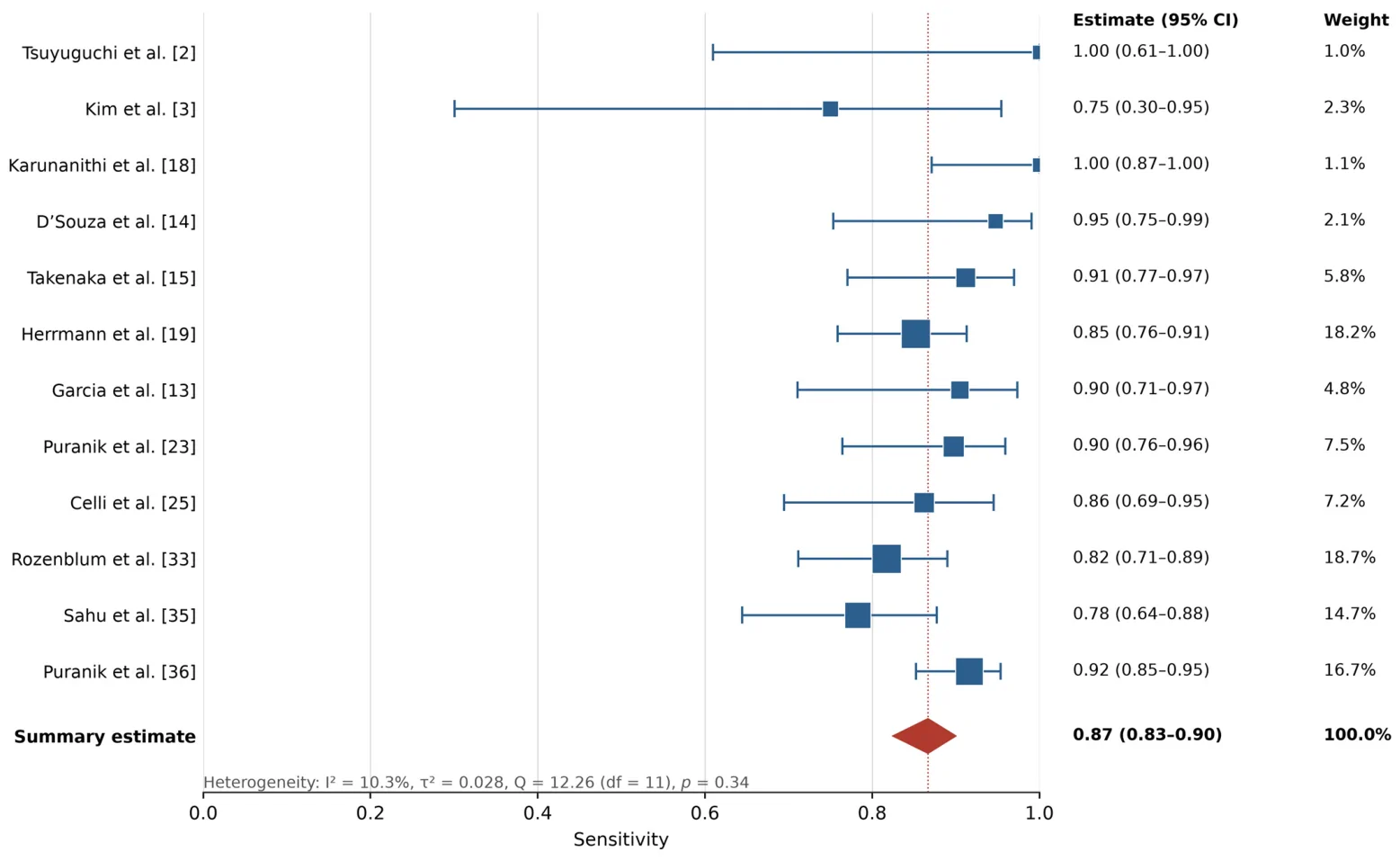
Forest plot of sensitivity for the 12 studies of the primary analysis. Circles are study estimates, sized in proportion to their weight in the random-effects model; horizontal lines are 95% Wilson score confidence intervals. The diamond is the summary estimate from the bivariate model. Heterogeneity: I^2^ = 10.3%, τ^2^ = 0.028, Q = 12.26 (df = 11), *p* = 0.34 [2,3,13,14,15,18,19,23,25,33,35,36].

**Figure 3:**
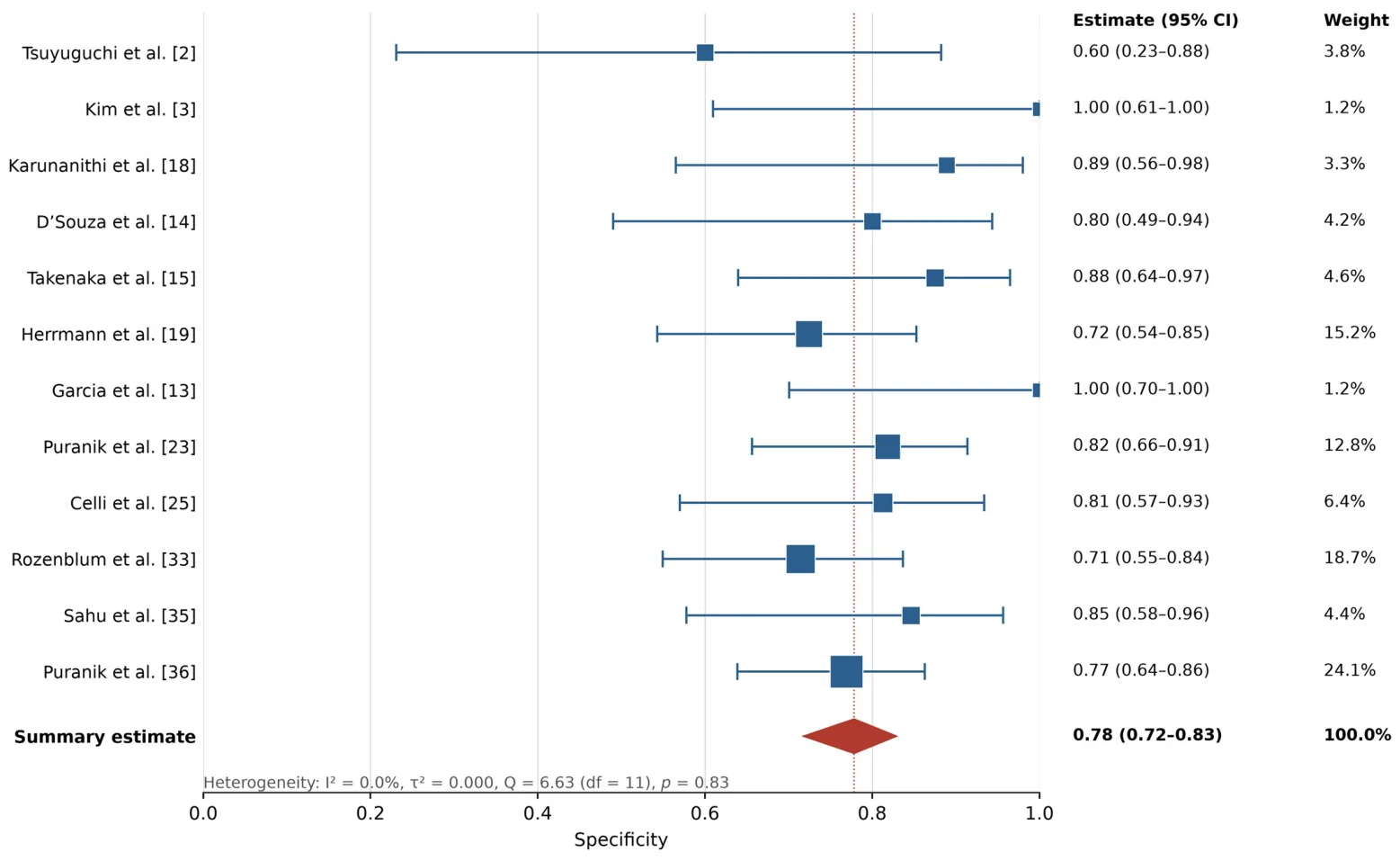
Forest plot of specificity for the 12 studies of the primary analysis, plotted as in Figure 2 with 95% Wilson score confidence intervals. Heterogeneity: I^2^ = 0%, τ^2^ = 0.000, Q = 6.63 (df = 11), *p* = 0.83. The apparent spread of point estimates reflects small denominators—eight studies contributed fewer than 20 patients without recurrence—rather than between-study variation [2,3,13,14,15,18,19,23,25,33,35,36].

**Figure 4:**
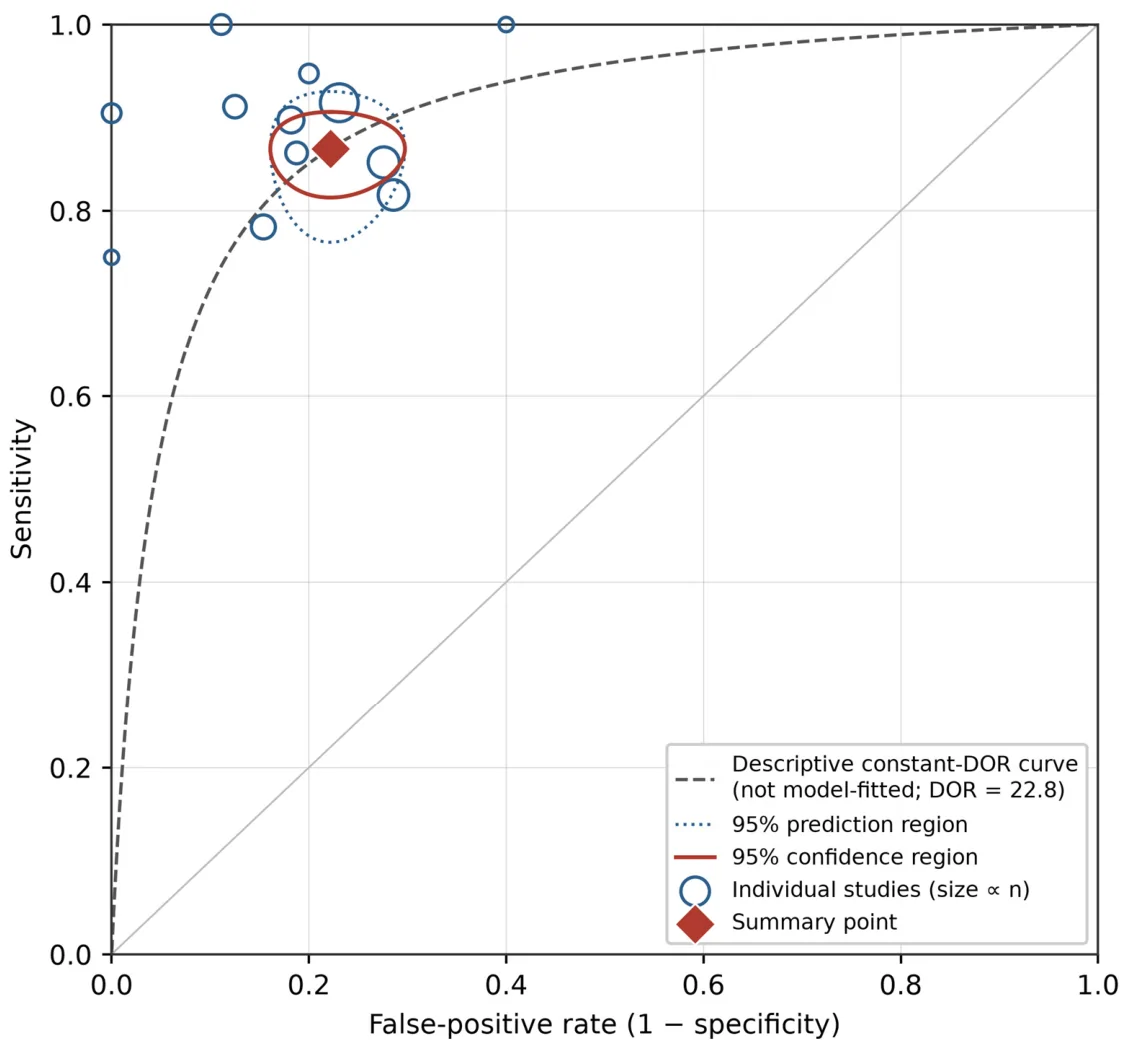
Receiver operating characteristic space for the 12 studies of the primary analysis. Circles represent individual studies, sized in proportion to sample size; the diamond is the summary point from the bivariate model, with its 95% confidence region (solid) and 95% prediction region (dotted). The prediction region is wider than the confidence region only in the sensitivity dimension, because no between-study variation in specificity was detectable. The dashed curve is a symmetric summary receiver operating characteristic approximation of constant diagnostic odds ratio (22.8; area under the curve 0.90). It is not a curve fitted to these data: because the between-study correlation was not estimable, no regression-based curve could be fitted, and the approximation is shown for comparability with earlier reviews only.

**Figure 5:**
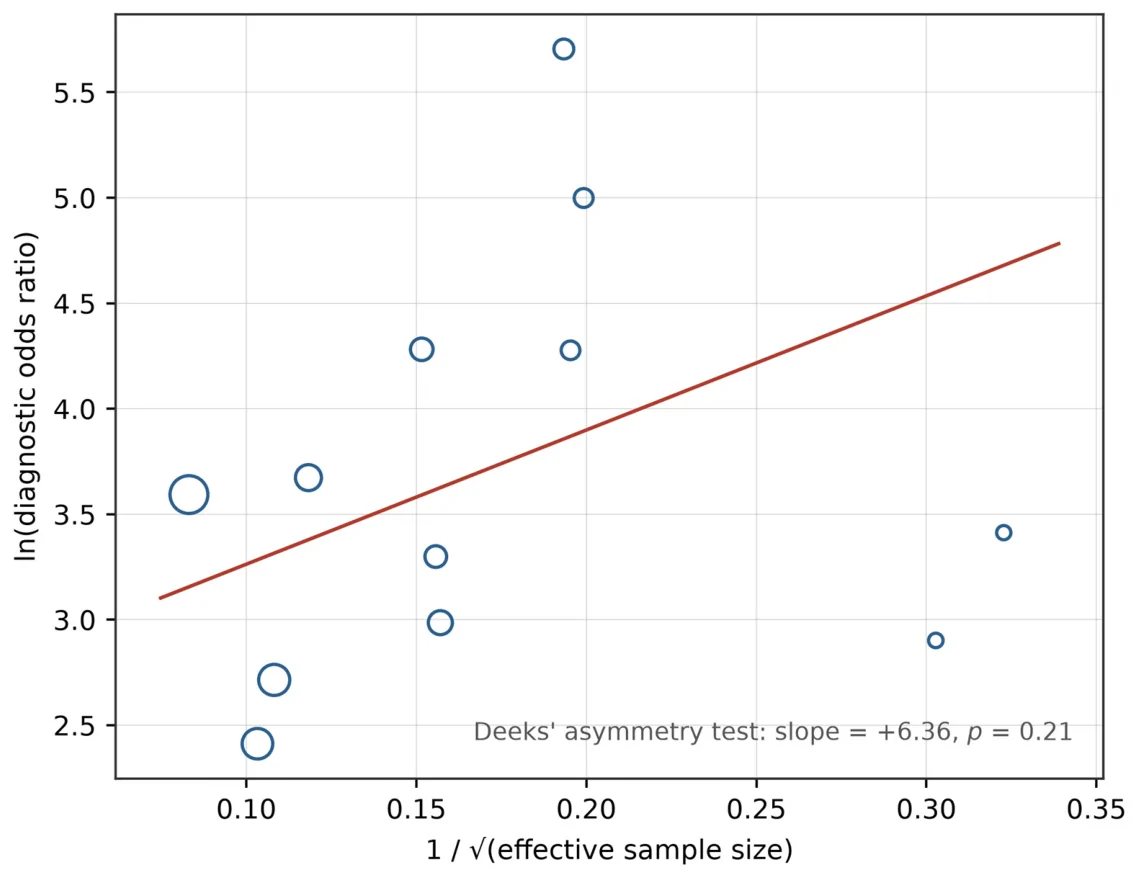
Deeks’ funnel plot for the 12 studies of the primary analysis: log diagnostic odds ratio against the inverse square root of the effective sample size, with the effective-sample-size-weighted regression line. Circle size is proportional to sample size. Slope +6.36, *p* = 0.21. With 12 studies, this test has very low power, and a non-significant result does not exclude small-study effects.

**Figure 6:**
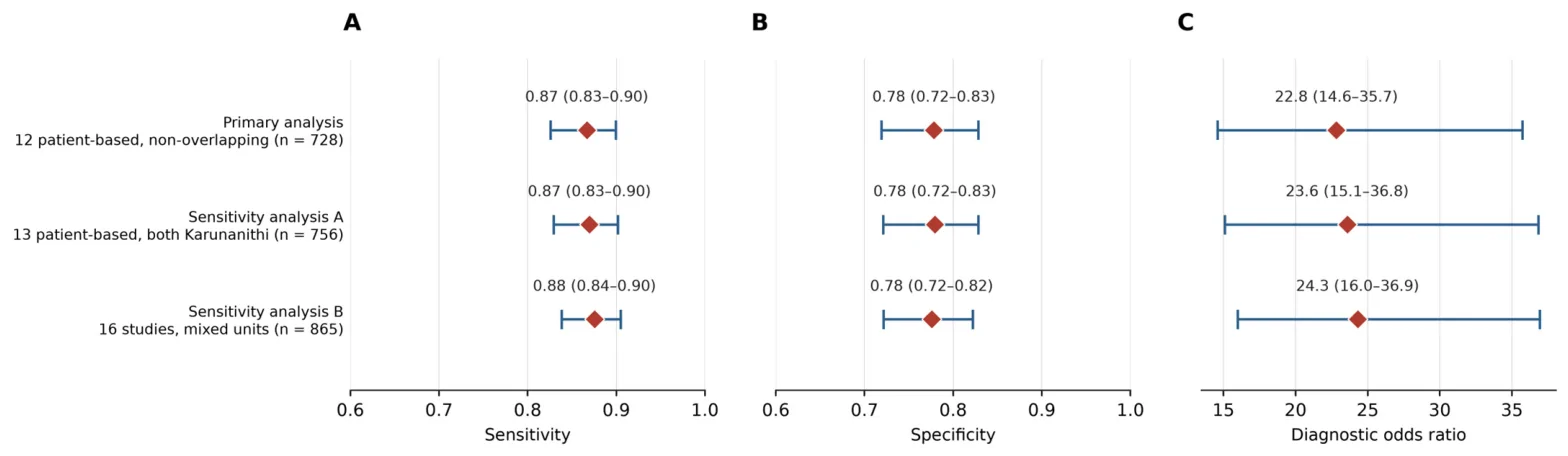
Forest plots and SROC representation for the sensitivity analysis, retaining both potentially overlapping Karunanithi datasets, for (**A**) sensitivity, (**B**) specificity, and (**C**) diagnostic odds ratio. Diamonds represent summary estimates, and bars represent 95% confidence intervals. Retaining both Karunanithi datasets (13 studies) produced estimates virtually identical to those of the primary analysis (12 studies).

**Figure 7:**
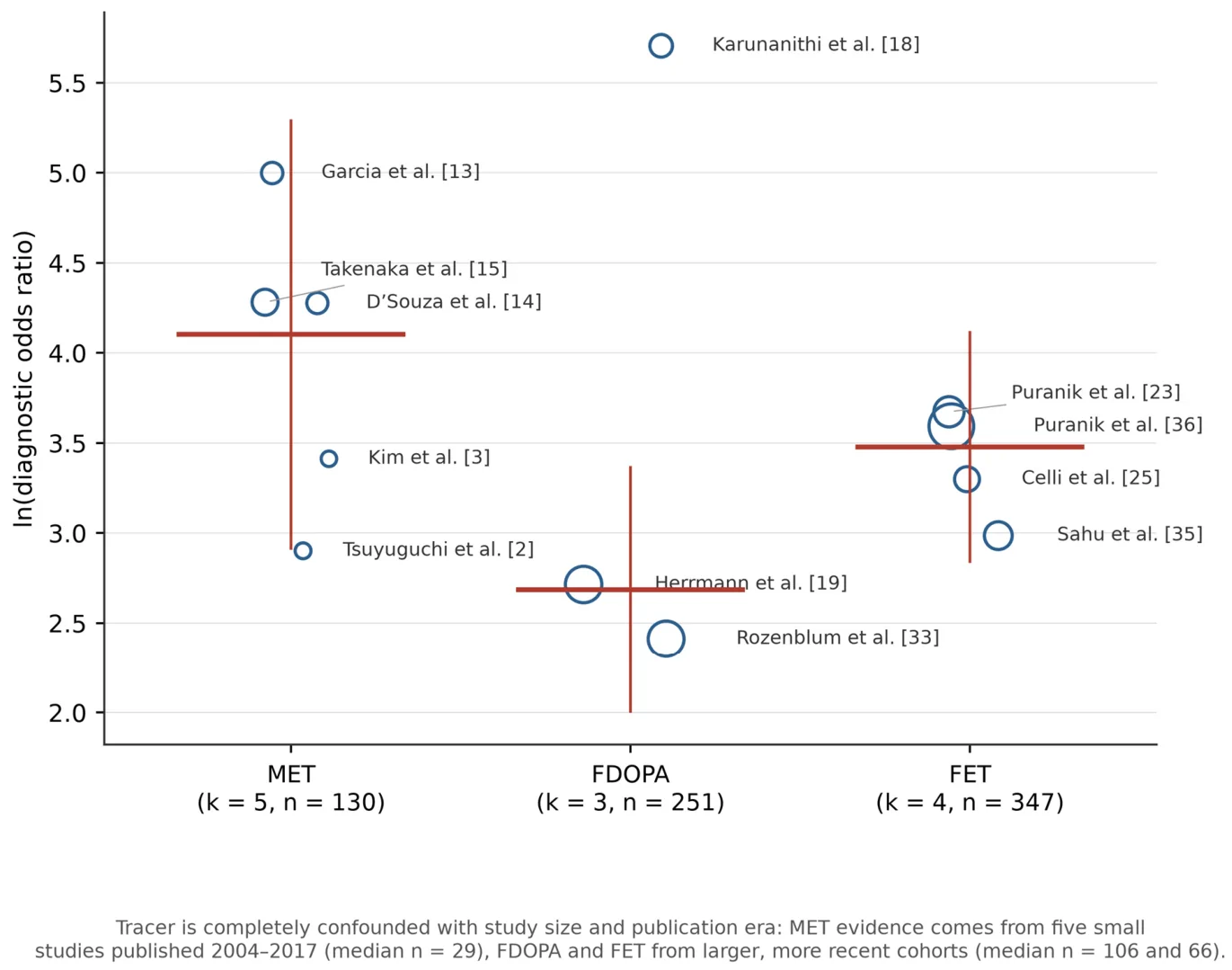
Log diagnostic odds ratio by tracer for the 12 studies of the primary analysis. Circles represent individual studies, sized in proportion to sample size; horizontal bars represent subgroup means with 95% confidence intervals. The apparent advantage of MET should not be interpreted as evidence of superior tracer performance, as tracer type is strongly confounded by publication era and study sample size. The five MET studies were published between 2004 and 2017 with a median of 29 patients each, whereas the FDOPA and FET studies are larger and more recent. No head-to-head within-patient comparison exists, and the observed differences cannot be attributed to true tracer-level effects [2,3,13,14,15,19,23,25,33,35,36].

**Table 1:** Characteristics of included studies and QUADAS-2 assessment.

No.	First Author (Year)	Country	Study Design	Unit of Analysis	Study Population	Tracer	Reference Standard	Patient Selection	Index Test	Reference Standard (RoB)	Flow and Timing	Follow-Up Duration/Criterion
1	Sonoda (1998) [1]	Japan	Retrospective	Scan-based	10 patients (12 PET examinations)	MET	Histopathology and/or clinical-radiological follow-up	H	U	U	U	3 months
2	Tsuyuguchi (2004) [2]	Japan	Retrospective	Patient-based	11 patients	MET	Histopathology and/or clinical-radiological follow-up	H	L	L	U	5 months
3	Kim (2010) [3]	Korea	Retrospective	Patient-based	10 patients	MET	Histopathology or serial clinical-radiological follow-up	H	U	U	U	12 months
4	Shishido (2012) [7]	Japan	Retrospective	Lesion-based	21 lesions	MET	Histopathology and/or clinical-radiological follow-up	H	U	L	U	2–70 months
5	Karunanithi (2013a) [17]	India	Prospective	Patient-based	28 patients	FDOPA	Histopathology and/or clinical-radiological follow-up	L	U	L	L	2–16 months
6	Karunanithi (2013b) [18]	India	Prospective	Patient-based	35 patients	FDOPA	Histopathology and/or clinical-radiological follow-up	L	U	L	L	12 months
7	D’Souza (2014) [14]	India	Retrospective	Patient-based	29 patients	MET	Histopathology and/or clinical-radiological follow-up	U	U	L	U	6 months
8	Takenaka (2014) [15]	Japan	Retrospective	Patient-based	50 patients	MET	Surgical histopathology	U	U	U	U	-
9	Herrmann (2014) [19]	USA	Retrospective	Patient-based	110 patients	FDOPA	Histopathology and/or clinical-radiological follow-up	U	H	U	U	6 months
10	Garcia (2017) [13]	Spain	Retrospective	Patient-based	30 patients	MET	Histopathology and/or clinical-radiological follow-up	U	U	L	U	6–12 months
11	Puranik (2021) [23]	India	Retrospective	Patient-based	72 patients	FET	Histopathology where available or multidisciplinary clinical and neuroimaging follow-up	U	H	U	U	3–13 months
12	Celli (2021) [25]	Italy	Retrospective	Patient-based	45 patients	FET	Histopathology and/or short-term MRI follow-up	U	H	U	U	1–1.5 months
13	Henriksen (2022) [29]	Denmark	Retrospective	Lesion-based	60 patients (76 lesions)	FET	Histopathology and/or clinical-radiological follow-up	U	U	L	U	6 months
14	Rozenblum (2023) [33]	France	Retrospective	Patient-based	106 patients	FDOPA	Histopathology and/or clinical-radiological follow-up	U	H	L	U	6 months
15	Sahu (2023) [35]	India	Retrospective	Patient-based	62 patients (59 evaluable)	FET	Histopathology and/or multidisciplinary clinical-radiological follow-up	U	U	U	U	2–84 months
16	Puranik (2025) [36]	India	Retrospective	Patient-based	171 patients	FET	Histopathology where available or multidisciplinary assessment based on serial imaging	U	U	L	U	6–12 months

Risk of bias was evaluated using the QUADAS-2 tool across four domains (patient selection, index test, reference standard, and flow and timing) and classified as low (L), high (H), or unclear (U). Unit of analysis indicates whether diagnostic accuracy was calculated on a patient-, lesion-, or scan-based dataset. Reference standards consisted of histopathological confirmation, clinical-radiological follow-up, or a combination of both, according to the individual study protocol. FU, follow-up; RoB, risk of bias.

**Table 2:** Summary of pooled diagnostic performance of amino acid PET for differentiating tumor recurrence from treatment-related changes. Values are presented with 95% confidence intervals (CI). PLR, NLR, and DOR were derived analytically from the fitted summary sensitivity and specificity estimates; their 95% confidence intervals were calculated using the delta method from the covariance matrix of the summary estimates.

Parameter	Value (95% CI)
Sensitivity	0.87 (0.83–0.90)
Specificity	0.78 (0.72–0.83)
PLR	3.91 (3.04–5.02)
NLR	0.17 (0.13–0.23)
DOR	22.8 (14.6–35.7)
AUC (SROC)	0.90 (symmetric SROC approximation)

**Table 3:** Summary diagnostic performance in the primary analysis and the sensitivity analysis retaining both potentially overlapping Karunanithi datasets.

Analysis	Studies	Diagnostic Observations	Sensitivity (95% CI)	Specificity (95% CI)	PLR	NLR	DOR (95% CI)
Primary analysis (patient-based, non-overlapping)	12	728	0.87 (0.83–0.90)	0.78 (0.72–0.83)	3.91	0.17	22.8 (14.6–35.7)
Sensitivity analysis A (potential cohort-overlap misclassification; both Karunanithi datasets retained)	13	756	0.87 (0.83–0.90)	0.78 (0.72–0.83)	3.94	0.17	23.6 (15.1–36.8)
One-stage binomial model, no continuity correction	12	728	0.88 (0.84–0.92)	0.79 (0.74–0.85)	4.29	0.15	29.1 (18.0–46.8)

PLR, positive likelihood ratio; NLR, negative likelihood ratio; DOR, diagnostic odds ratio.

**Table 4:** Subgroup analysis of diagnostic performance according to tracer type, primary analysis (12 studies).

Subgroup	Sensitivity (95% CI)	Specificity (95% CI)
FET PET (k = 4, n = 347)	0.87 (0.79–0.92)	0.80 (0.71–0.86)
MET PET (k = 5, n = 130)	0.89 (0.80–0.95)	0.82 (0.67–0.91)
FDOPA PET (k = 3, n = 251)	0.84 (0.78–0.89)	0.73 (0.62–0.82)

Note: Observed differences between tracer subgroups should not be interpreted as evidence of differential tracer performance, as tracer type was strongly confounded by publication era and study sample size, and no head-to-head within-patient comparisons were available.

## Data Availability

All data analyzed in this systematic review and meta-analysis were obtained from previously published studies and are available in the corresponding publications and Supplementary Materials. No new patient-level data were generated or collected for this study.

## Supplementary Materials

The following supporting information can be downloaded at: https://www.mdpi.com/article/10.3390/cancers18193069/s1, Supplementary Table S1. Study-level diagnostic datasets included in the quantitative synthesis. Prisma Checklist. Complete Code Analysis.
