# Supplementary material for: Diagnostic Accuracy of Amino Acid PET for Differentiating Tumor Recurrence or Progression from Treatment-Related Changes in Glioma Patients: A Systematic Review and Meta-Analysis
Source: Cancers (Basel). 2026 Sep 22;18(19):3069. doi: 10.3390/cancers18193069 (PMC13664548; doi:10.3390/cancers18193069)
Supplement: Supplementary file 1 [file cancers-18-03069-s001.zip › Supplementary_table_05.08.26.pdf]

Supplementary Table S1. Study-level diagnostic datasets included in the quantitative synthesis.

No	Study	Year	Country	TP	FP	FN	TN	Enrolled patients (n)	Diagnostic observations in 2×2 (n)	Recurrence_n	NonRecurrence_n	Tracer	Design	Reference	PET type	Analysis	Tumor grade	SUV metric
1	Sonoda 1998	1998	Japan	5	1	0	6	10	12	5	7	MET	retrospective	mixed	static	scan-based	mixed	T/N ratio
2	Tsuyuguchi 2004	2004	Japan	6	2	0	3	11	11	6	5	MET	retrospective	mixed	static	patient	HGG	visual
3	Kim 2010	2010	Korea	3	0	1	6	10	10	4	6	MET	retrospective	mixed	static	patient	HGG	Lmax/Rmax
4	Shishido 2012	2012	Japan	15	5	0	1	21	21	15	6	MET	retrospective	mixed	static	lesion-based	HGG	L/N ratio
5	Karunanithi 2013a	2013	India	21	1	0	6	28	28	21	7	FDOPA	prospective	mixed	static	patient	mixed	SUV
6	Karunanithi 2013b	2013	India	26	1	0	8	35	35	26	9	FDOPA	prospective	mixed	static	patient	mixed	SUV
7	D'Souza 2014	2014	India	18	2	1	8	29	29	19	10	MET	retrospective	mixed	static	patient	HGG	L/N ratio
8	Takenaka 2014	2014	Japan	31	2	3	14	50	50	34	16	MET	retrospective	histopathology	static	patient	HGG	L/N ratio
9	Herrmann 2014	2014	USA	69	8	12	21	110	110	81	29	FDOPA	retrospective	mixed	static	patient	GBM	L/S ratio
10	Garcia 2016	2016	Spain	19	0	2	9	30	30	21	9	MET	retrospective	mixed	static	patient	HGG	SUV ratio
11	Puranik 2021	2021	India	35	6	4	27	72	72	39	33	FET	retrospective	mixed	static	patient	HGG	T/W ratio
12	Celli 2021	2021	Italy	25	3	4	13	45	45	29	16	FET	retrospective	mixed	dynamic	patient	mixed	TBRmax
13	Henriksen 2022	2022	Denmark	40	5	4	27	60	76	44	32	FET	retrospective	mixed	static	lesion-based	HGG	TBRmax
14	Rozenblum 2023	2023	France	58	10	13	25	106	106	71	35	FDOPA	retrospective	mixed	static+dynamic	patient	HGG	TBRmean

No	Study	Year	Country	TP	FP	FN	TN	Enrolled patients (n)	Diagnostic observations in 2×2 (n)	Recurrence_n	NonRecurrence_n	Tracer	Design	Reference	PET type	Analysis	Tumor grade	SUV metric
15	Sahu 2023	2023	India	36	2	10	11	62	59	46	13	FET	retrospective	mixed	static+MRI	patient	HGG	T/W ratio
16	Puranik 2025	2025	India	109	12	10	40	171	171	119	52	FET	retrospective	mixed	static	patient	HGG	TBR

The number of diagnostic observations included in the 2 × 2 contingency tables may differ from the number of enrolled patients in studies using lesion-based or scan-based analyses or excluding non-evaluable cases.
